# Acceptability of flavoured pharmaceutically non-active mini-tablets in pet cats tested with a rapid 3-portal acceptance test with and without food

**DOI:** 10.1016/j.vas.2019.100054

**Published:** 2019-03-01

**Authors:** S. Savolainen, J. Hautala, J. Junnila, S. Airaksinen, A.M. Juppo, M. Raekallio, O. Vainio

**Affiliations:** a Department of Equine and Small Animal Medicine, University of Helsinki, P.O. Box 57, 00014 University of Helsinki, Finland; b Division of Pharmaceutical Chemistry and Technology, University of Helsinki, P.O. Box 56, 00014 University of Helsinki, Finland; c 4Pharma Ltd, Arkadiankatu 7, 00100 Helsinki, Finland

**Keywords:** Cat, Amino acids, Synthetic flavours, Drug development, Palatability testing

## Abstract

Palatable oral pharmaceuticals are crucial for feline medication. The pharmaceutical industry prefers synthetic flavours over organic ones because of hygiene and regulatory issues. The aim of this study was to find a palatable synthetic flavour for future taste-masking of feline pharmaceuticals. The hypothesis was that synthetic meat aromas and free amino acids would be palatable to cats. The palatability of 18 synthetically flavoured mini-tablets was screened with 10–19 pet cats using a rapid 3-portal acceptance test with and without food. The tested flavours were synthetic amino acids (L-carnitine, l-glutamic acid monosodium salt hydrate, l-leucine, l-methionine, l-phenylalanine, l-proline, and taurine), d-(+)-Maltose monohydrate and thiamine hydrochloride. Furthermore, thiamine hydrochloride was combined with amino acids (l-cysteine, l-leucine, l-methionine and l-proline) and synthetic meat flavours (2-acetylpyridine, 2-acetylthiazole, 2-pentylpyridine and 4-hydroxy-5-methyl-3(*2H*)-furanone). The negative control was a non-flavoured placebo mini-tablet, while positive controls were an organic yeast-flavoured mini-tablet and a yeast- and fish-based commercial vitamin tablet in mini-tablet form. No significant differences were detected between palatable synthetic flavours and the placebo, nor between the synthetic flavours and the yeast flavour. In general, the mini-tablet seemed to be small enough to be accepted inside a food item. These results differ from the earlier literature about the taste preferences of cats for amino acids, and hence free amino acids should not be considered palatable to cats based purely on previous findings.

## Highlights

• The palatability of synthetically flavoured mini-tablets in cats was investigated.
• 3-portal acceptance tests were carried out on 10–19 pet cats in their homes.
• The mini-tablets were not accepted voluntarily without food by most of the cats.
• Amino acids were not palatable to cats, which did not support the earlier studies.
• Most of the cats ate the mini-tablets concealed inside a palatable food item.

## 1 Introduction

Cats can be difficult to medicate against their will (Thombre, 2004). Forcing a cat to take a pill can lead to owner injuries and also have a negative effect on the cat-owner relationship. Most of the problems concerning feline medication are related to unpleasant taste and many of the off-label solid pharmaceuticals are too large in size for cats (Sivén et al., 2016). Voluntary acceptance of the pharmaceutical would likely increase the medication adherence in cats, and improve the success of the treatment (Thombre, 2004). Hence, there is a need for palatable and easily administered feline pharmaceuticals.

The most preferred flavour would be synthetic, because organic flavours complicate the manufacturing process, and regulatory issues for meat-based flavours may also exist (Ahmed and Kasraian, 2002, Thombre, 2004). However, cats can be selective over their food (Bradshaw, Healey, Thorne, Macdonald, & Arden-Clark, 2000), and therefore finding a palatable flavour acceptable to most cats is challenging.

In the literature reviewed, cats are widely considered to prefer certain amino acids (Bradshaw, 1991, Bradshaw et al., 1996, MacDonald et al., 1984, Thombre, 2004, Zaghini and Biagi, 2005). However, this information is based on old neurophysiological studies in anesthetized cats, where the sensory effect of chemical substances was studied (Boudreau, 1974, Boudreau and Alev, 1973), and preference studies carried out on laboratory cats (Beauchamp et al., 1977, White and Boudreau, 1975). l-proline, l-cysteine, l-ornithine, l-lysine, l-histidine and l-alanine triggered a discharge in the amino acid units on the tongue of the cat, while l-tryptophan, l-isoleucine, l-arginine and l-phenylalanine inhibited the units (Boudreau, 1974, Boudreau and Alev, 1973). Interestingly, the triggering amino acids have been described as ‘sweet’, and the inhibiting ones as ‘bitter’ by humans (Boudreau, Oravec, & White, 1981). When tested as saline or water solutions, laboratory cats preferred l-proline, l-lysine and l-histidine, and avoided l-tryptophan, l-isoleucine, adenine and l-glutamic acid, preferred or avoided l-alanine, and neither preferred nor avoided glycine (Beauchamp et al., 1977, White and Boudreau, 1975). Laboratory kittens chose food containing large quantities of leucine (Hargrove, Morris, & Rogers, 1994). Various methods have been used to assess the palatability of feline pharmaceuticals (Bernachon et al., 2014a, Bernachon et al., 2014b, Cron et al., 2014, Giraudel et al., 2010, Gunew et al., 2008, Huhtinen et al., 2015, Khor et al., 2011, Litster et al., 2007, Morton et al., 2011, Traas et al., 2010) because a guideline on the demonstration of palatability of veterinary medicinal products has only been published quite recently by the European Medicines Agency (EMA, 2014).

The primary aim of our study was to find a palatable synthetic flavour for future taste- masking of feline pharmaceuticals by screening flavours compressed into a mini-tablet form with a rapid 3-portal acceptance test on pet cats. Secondary aims were to evaluate the acceptability of a mini-tablet when concealed inside a palatable food item, and to test the feasibility of an owner-performed 3-portal acceptance test carried out on pet cats in their home environment.

The hypotheses were that palatable flavours for cats exist among the tested flavours, and that the size of a mini-tablet would be ideal when administered inside a palatable food item. Other hypotheses were that a commercial vitamin tablet and an organic yeast flavour would be palatable for cats in mini-tablet form.

## 2 Material and methods

The Research Ethics Committee of the University of Helsinki approved the study protocol on 18 November 2010.

### 2.1 Cats

A total of 23 adult cats belonging to 10 owners participated in the seven trials. Ten cats were enrolled in most of the trials, but trial 3 included 19 cats (supplementary Table S1). Most of the cats were castrated males (*n* = 15), five cats were spayed females, two were intact females and one was an intact male. The age of the cats varied from one to 15 years at the time of the study, and most of the cats did not have free access to outdoors, only four of the cats were able to go outdoors freely. The owners were recruited among the students and personnel of the Faculty of Veterinary Medicine at the University of Helsinki, as well among their friends. No restrictions were placed on the age or breed of the cats, or on the number of cats in the households. According to the owners, the cats were healthy and had a normal appetite. Participation was voluntary, and the owners did not receive any financial inducements. All of the owners signed consent forms.

### 2.2 Tested items

The synthetically flavoured mini-tablets (25 mg) consisted of the tested flavours and tablet excipients. They were round with a biconvex shape and a diameter of 3 mm. We also tested the acceptability of a non-flavoured mini-tablet (placebo), consisting of tablet excipients, inside a food item. Positive controls comprised an organic yeast-flavoured mini-tablet (Yeast Extract, AppliChem) and a commercial vitamin tablet (Kitzyme, Bob Martin) in mini-tablet form. The commercial vitamin tablet was also tested in its original form to ensure the acceptable mouthfeel of the mini-tablet. The ingredients and the manufacturers are listed in Table 1, and the compositions of the mini-tablets in Table 2.

**Table 1:** Ingredients used in the study.

Ingredients used		Manufacturer
Excipient	Microcrystalline cellulose (Avicel PH 101)	FMC Corporation, Ireland
	Mannitol (Pearlitol 160 C)	Roquette, France
	Hydroxypropyl cellulose Sodium stearyl fumarate (Pruv)	Aqualon France SA, France JRS Pharma, Spain
	Microcrystalline cellulose (Emcocel LP200)	JRS Pharma, Germany
Flavour	l-carnitine	Sigma-Aldrich, USA
	l-cysteine	Sigma-Aldrich, Japan
	l-glutamic acid monosodium salt hydrate	Sigma-Aldrich, USA
	l-leucine	Sigma-Aldrich, Japan
	l-methionine	Sigma-Aldrich, Japan
	l-proline	Sigma-Aldrich, USA
	l-phenylalanine	Sigma-Aldrich, USA
	Taurine	Sigma-Aldrich, USA
	Thiamine hydrochloride	Hawkins Inc., USA
	d-(+)-maltose monohydrate	Sigma-Aldrich, USA
	2-acetylthiazole	Sigma-Aldrich, China
	2-acetylpyridine	Sigma-Aldrich, China
	4‑hydroxy-5-methyl-3(*2H*)-furanone	Sigma-Aldrich, USA
	2-pentylpyridine	Sigma-Aldrich, USA
	Yeast extract	AppliChem, Germany
Vitamin tablet	Commercial vitamin tablet for cats (Kitzyme)	Bob Martin, UK

**Table 2:** Trial number, number of cats, flavours of tested mini-tablets and tablet composition.

Trial number	Number of tested cats	Tablet flavour(s) and amount in tablet composition (% m/m)	Tablet excipient(s) and amount in tablet composition (% m/m)
1	10	Non-flavoured placebo	Microcrystalline cellulose (Avicel PH 101) (70% m/m)
			Mannitol (Pearlitol 160C) (25% m/m) Hydroxypropyl cellulose (5% m/m)
			Sodium stearyl fumarate (Pruv) (1% m/m)
2	10	l-glutamic acid monosodium salt hydrate, 50% m/m	Microcrystalline cellulose (Emcocel LP200), 50% m/m
		l-leucine, 50% m/m	Microcrystalline cellulose (Emcocel LP200), 50% m/m
		l-methionine, 50% m/m	Microcrystalline cellulose (Emcocel LP200), 50% m/m
		l-phenylalanine, 50% m/m	Microcrystalline cellulose (Emcocel LP200), 50% m/m
		l-proline, 50% m/m	Microcrystalline cellulose (Emcocel LP200), 50% m/m
		Thiamine hydrochloride, 50% m/m	Microcrystalline cellulose (Emcocel LP200), 50% m/m
3	19	Thiamine hydrochloride, 50% m/m	Microcrystalline cellulose (Emcocel LP200), 50% m/m
		Thiamine hydrochloride - l-cysteine, 50% m/m of flavour 1:1 mixture	Microcrystalline cellulose (Emcocel LP200), 50% m/m
		Thiamine hydrochloride - l-leucine, 50% m/m of flavour 1:1 mixture	Microcrystalline cellulose (Emcocel LP200), 50% m/m
		Thiamine hydrochloride - l-methionine, 50% m/m of flavour 1:1 mixture	Microcrystalline cellulose (Emcocel LP200), 50% m/m
		Thiamine hydrochloride -l-proline, 50% m/m of flavour 1:1 mixture	Microcrystalline cellulose (Emcocel LP200), 50% m/m
4	10	l-carnitine, 50% m/m	Microcrystalline cellulose (Emcocel LP200), 50% m/m
		Taurine, 50% m/m	Microcrystalline cellulose (Emcocel LP200), 50% m/m
5	10	d-(+)-maltose monohydrate, 50% m/m	Microcrystalline cellulose (Emcocel LP200), 50% m/m
6	10	2-acetylthiazole, 2% m/m	Microcrystalline cellulose (Emcocel LP200), 98% m/m
		2-acetylpyridine, 2% m/m	Microcrystalline cellulose (Emcocel LP200), 98% m/m
		4‑hydroxy-5-methyl-3(*2H*)-furanone, 2% m/m	Microcrystalline cellulose (Emcocel LP200), 98% m/m
		2-pentylpyridine, 2% m/m	Microcrystalline cellulose (Emcocel LP200), 98% m/m
7	10	Yeast extract, 50% m/m	Microcrystalline cellulose (Emcocel LP200), 50% m/m
		Commercial vitamin tablet (Kitzyme, Bob Martin, UK) in mini-tablets containing the following ingredients: dried brewer's yeast, vegetable protein, dicalcium phosphate, encapsulated fish oil, calcium carbonate, silicon dioxide, magnesium stearate, fish powder	-

### 2.3 Study design

The owners were blinded to the tested items, and the mini-tablets were offered in a random order to the cat, with the exception of trial 5, where only one mini-tablet was tested. The mini-tablets were packed in small transparent plastic bags. The bags were numbered manually with a pen, and the tablets were tested in numerical order.

The trials were carried out in the home environments of the cats by the owners, and the researchers were not present in the test situation. The owners were instructed in writing about the study protocol and to perform the palatability trials when the cat was cooperative and alert. A table was recommended as a suitable testing area. The type of food was chosen by the owners based on the individual taste preferences of each cat. The food item was supposed to be small enough to be eaten at one go (supplementary Table S1). The cats were familiarized with the test situation by offering food on the table. To standardize the hunger status of the cats, they were not fed for at least six hours before the trial or, if fed *ad libitum*, overnight. The cats were not forced to participate in the trials; they were lifted onto the table if necessary, after which they were free to choose their actions.

The mini-tablets were tested by using a rapid 3-portal acceptance test as follows: first, the cat was offered the mini-tablet on the table. If the cat did not notice the mini-tablet, the owner pointed to it with a finger. If the cat did not eat the mini-tablet (ate: yes/no), it was then offered by hand, holding it between two fingers, so that the cat could sniff the tablet. If the cat did not accept the tablet (ate: yes/no), it was finally provided by concealing it in the palatable food item (ate: yes/no). The owners were also able to write down comments.

In most trials, only one mini-tablet was tested per day, but if the cat was cooperative, one or two mini-tablets could be tested on the same day in trial 3 (Table 2), and in trial 1 three similar placebo mini-tablets were tested on the same or consecutive days, and only inside a palatable food item.

### 2.4 Statistical methods

Due to the low number of cats, the analysis was mainly descriptive. One-way frequency tables were generated for each mini-tablet type: synthetic flavours, positive controls and placebo (Table 3). The synthetically flavoured mini-tablets and the two controls (the yeast-flavoured and the commercial vitamin-flavoured mini-tablets) were compared descriptively with cross tabulations. Dichotomized variables were used for cross tabulations of the synthetic flavours against the placebo (ate vs. did not eat the tablet). The synthetically flavoured mini-tablets were compared with the yeast-flavoured and the commercial vitamin-flavoured mini-tablets using Wilcoxon signed-rank tests. All statistical analyses were performed using SAS System for Windows, version 9.3 (SAS Institute Inc., Cary, NC, USA). *P* ≤ 0.05 was considered to be statistically significant, and *P* > 0.05 to *P* ≤ 0.10 was considered to show a tendency.

**Table 3:** Acceptance and interest towards each flavoured mini-tablet separately. NT=not tested.

Flavoured mini-tablet	Ate the tablet from the table n (%)	Ate the tablet from hand n (%)	Ate the tablet concealed inside a food itemn (%)	Did not eat the tablet at all n (%)	Took the tablet into the mouth and dropped it n (%)	Licked the tablet n (%)
Placebo^a^^,^^b^	NT	NT	8 (88.9)	1 (11.1)	NT	NT
l-glutamic acid monosodium salt hydrate	0 (0.00)	0 (0.00)	9 (90.0)	1 (10.0)	0 (0.00)	0 (0.00)
l-leucine	0 (0.00)	1 (10.0)	8 (80.0)	1 (10.0)	0 (0.00)	0 (0.00)
l-methionine	0 (0.00)	0 (0.00)	9 (90.0)	1 (10.0)	1 (10.0)	0 (0.00)
l-phenylalanine^c^	0 (0.00)	0 (0.00)	8 (88.9)	1 (11.1)	0 (0.00)	0 (0.00)
l-proline	0 (0.00)	0 (0.00)	8 (80.0)	2 (20.0)	1 (10.0)	1 (10.0)
Thiamine hydrochloride^d^	0 (0.00)	0 (0.00)	8 (88.9)	1 (11.1)	2 (22.2)	1 (11.1)
Thiamine hydrochloride	1 (5.30)	1 (5.30)	14 (73.7)	3 (15.8)	3 (15.8)	3 (15.8.)
Thiamine hydrochloride - l-cysteine	1 (5.30)	1 (5.30)	14 (73.7)	3 (15.8)	0 (0.00)	5 (26.3)
Thiamine hydrochloride - l-leucine	0 (0.00)	2 (10.5)	13 (68.4)	4 (21.1)	3 (15.8)	0 (0.00)
Thiamine hydrochloride - l-methionine	1 (5.30)	0 (0.00)	16 (84.2)	2 (10.5)	1 (5.30)	0 (0.00)
Thiamine hydrochloride - l-proline	2 (10.5)	0 (0.00)	15 (78.9)	2 (10.5)	1 (5.30)	0 (0.00)
l-carnitine	1 (10.0)	0 (0.00)	9 (90.0)	0 (0.00)	0 (0.00)	0 (0.00)
Taurine	0 (0.00)	0 (0.00)	9 (90.0)	1 (10.0)	0 (0.00)	0 (0.00)
d-(+)-Maltose monohydrate	0 (0.00)	0 (0.00)	9 (90.0)	1 (10.0)	0 (0.00)	0 (0.00)
2-acetylthiazole	0 (0.00)	0 (0.00)	7 (70.0)	3 (30.0)	0 (0.00)	0 (0.00)
2-acetylpyridine	0 (0.00)	0 (0.00)	6 (60.0)	4 (40.0)	0 (0.00)	1 (10.0)
4‑hydroxy-5-methyl-3*(2H)*-furanone	0 (0.00)	0 (0.00)	7 (70.0)	3 (30.0)	0 (0.00)	1 (10.0)
2-pentylpyridine	0 (0.00)	0 (0.00)	5 (50.0)	5 (50.0)	0 (0.00)	0 (0.00)
Commercial vitamin tablet, original^e^	5 (50.0)	0 (0.00)	NT	5 (50.0)	0 (0.00)	0 (0.00)
Commercial vitamin tablet, mini-tablet	5 (50.0)	0 (0.00)	5 (50.0)	0 (0.00)	0 (0.00)	0 (0.00)
Yeast extract	4 (40.0)	0 (0.00)	6 (60.0)	0 (0.00)	1 (10.0)	2 (20.0)

a Tested three times, only concealed inside a food item.

b One cat excluded because of discrepancy between results of the three trials (ate once with a food item).

c Tested twice on the same cat because of owner confusion.

d Tested twice on the same cat because of owner confusion.

e Tested without a food item.

## 3 Results

The acceptance of each flavoured mini-tablet is presented in Table 3. No synthetic flavour was significantly more acceptable than the placebo mini-tablet when administered inside a food item. No statistically significant differences were detected between the synthetic-flavoured mini-tablets and the yeast-flavoured mini-tablet, but the commercial vitamin-flavoured mini-tablet tended to be more palatable than most of the synthetically flavoured mini-tablets (*P* = 0.0625). The same five cats ate the commercial vitamin-flavoured mini-tablets and the original commercial vitamin tablets, while the other five cats ate neither of them.

While cats did not eat the synthetically flavoured mini-tablets without food, the owners commented that some of the cats showed interest towards them (Table 3). One owner commented that the size of the mini-tablet was optimal. Another mentioned that the small-sized tablet might be difficult for the cat to notice. One owner wrote that the mini-tablet stuck to the cat's nose while the cat was sniffing it. The owners commented in trial 3 that one cat was not motivated by the food, while another was used to receiving treats by hand. In trial 6, four cats were not motivated by the food in some of the tests. One owner stated that her cat ate the l-carnitine mini-tablet from the table, but vomited immediately and showed signs of short-term nausea. No other side effects were observed during the trials.

## 4 Discussion

The original purpose of this study was to rapidly screen the acceptability of several synthetic flavours pending further testing with preference tests to find the most palatable flavour for the taste- masking of feline pharmaceuticals. The main finding was that amino acids did not prove to be palatable to cats, which contradicted the previous studies (Beauchamp et al., 1977, Boudreau, 1974, Boudreau and Alev, 1973, White and Boudreau, 1975). Although the number of cats tested was low, the results seemed evident: most of the cats did not voluntarily accept the synthetically flavoured mini-tablets without them being concealed inside a palatable food item. On the other hand, the mini-tablet form appeared ideal for concealment in a food item, and its mouthfeel seemed to be acceptable to the cats. Furthermore, we observed that owner-perceived palatability trials carried out on pet cats in their home environments could be used for testing the palatability of pharmaceuticals for cats.

Amino acids were chosen for test purposes because of the carnivorous nature of cats, as amino acids are precursors of meat. In our study, most of the cats did not voluntarily accept the tested amino acids without food. Even though we did not test the palatability of the same quantity of amino acids as in earlier studies, our results suggest that free amino acids should not be claimed to be palatable to cats based solely on previous studies without further palatability testing.

Thiamine hydrochloride is a major component of yeast, and also a meat flavour precursor (Varavinit, Shobsngob, Bhidyachakorawat, & Subhantrika, 2000). Overall, most of the cats ate these flavoured mini-tablets only when concealed in a palatable food item. Thiamine tastes bitter to humans (Stacey & Sullivan, 2003), and cats are very sensitive to bitter taste (Lei et al., 2015), which may explain the low voluntary acceptance of thiamine and its combinations with amino acids. Approximately 25% of the cats showed interest towards the mini-tablet of thiamine hydrochloride combined with l-cysteine, a meat flavour precursor (Varavinit et al., 2000), by licking it. However, this cannot necessarily be construed as a positive reaction, because licking food has been related to a less palatable taste (Van den Bos, Meijer, & Spruijt, 2000). Unfortunately, we did not test the palatability of l-cysteine alone.

D-(+)-Maltose monohydrate is the predominant sugar in malt extract, which is commonly used in Cat Malt products as an appetite stimulant. However, it was not found to be palatable in our trial. On the other hand, it is detected through T1R2 and T1R3 sweet taste receptors (Pullicin, Penner, & Lim, 2017), and cats genetically lack the functional T1R2 receptor (Li et al., 2005).

None of the cats consumed the synthetic meat-flavoured mini-tablets without them being concealed inside a food item, and some did not eat them even when concealed in this way. Possibly their smell, taste or both were unpleasant, but on the other hand, according to the owners, some of the cats were not motivated to eat even the palatable food. Some unspecified synthetic meat flavours have been used in feline pharmaceuticals, which did not have complete voluntary consumption success either (Bernachon et al., 2014a, Huhtinen et al., 2015).

Microcrystalline cellulose was chosen for excipient of the mini-tablets based on that it is harmless to cats, and also it is considered to be tasteless to humans (Ejikeme, 2008), but this is not verified in cats. Most of the flavoured mini-tablets consisted of 50% of the flavour and 50% of microcrystalline cellulose, but the synthetic meat flavours had a very strong aroma even to the human nose, and therefore synthetic meat-flavoured mini-tablets consisted of 2% of the flavour and 98% of microcrystalline cellulose to reduce the strong aroma.

The small-sized mini-tablets were generally well accepted when concealed inside a palatable food item. However, as the food was not standardized, the mini-tablets may have dropped out of some food types more easily. In our previous study, the cats dropped a food item containing a similar mini-tablet significantly more often than food without the tablet (Savolainen et al., 2016). It would be recommendable, therefore, to use standardized food to prevent the consistency of the food from having an impact. However, the acceptability of the food should be verified before the trials. It is possible that the food was not palatable enough because some owners mentioned that the cat did not eat the food in every test.

According to the EMA guideline (EMA, 2014), a pharmaceutical can be claimed to be palatable if at least 70% of cats eat it voluntarily without food; hence, the flavoured mini-tablets could not be considered palatable to the cats, not even in the case of our positive controls. In our trials, a few cats took the mini-tablet into their mouth but dropped it, and some cats licked it without eating it. In one palatability study of anti-parasitic drugs carried out on laboratory cats, prehension from a bowl or hand was assessed in addition to consuming the product (Bernachon et al., 2014a). Prehension was defined as the cat voluntarily taking the tablet into their mouth, even if it was not swallowed. The researchers discovered high prehension numbers in adult cats compared to our pet cats, but the consumption was much lower (Bernachon et al., 2014a). However, the behaviour and taste preferences of pet and laboratory cat populations differ (Bradshaw et al., 2000). Pet cats live in more enriched environments compared to laboratory cats, and also eat a more varied diet (Laflamme et al., 2008).

The cats in our study had variation in their sex, age and breed, and also in their lifestyles as four of them had also access to go freely outdoors. On the other hand, despite of the high individual variation, using pet cats is consistent with the EMA guideline (EMA, 2014) for carrying out palatability tests for pharmaceuticals on companion animals with the target population. Some palatability assessments of pharmaceuticals have been conducted on client-owned pet cats (Giraudel et al., 2010, Gunew et al., 2008, Huhtinen et al., 2015, Litster et al., 2007, Morton et al., 2011), and others on laboratory cats (Bernachon et al., 2014a, Bernachon et al., 2014b, Cron et al., 2014). Cats may have a timid nature and a fear of strangers or unfamiliar environments, and therefore owner-performed palatability testing would help to avoid stress, and thereby obtain more reliable results. However, care should be taken that owners are well instructed before the trial. In our study, most of the owners followed the instructions correctly. Tests carried out by the owner could also be recorded with a video camera for later analysis, as we did in our previous study in the feeding situation (Savolainen et al., 2016). In our trials, the palatability was evaluated by the voluntary acceptance of the mini-tablet. In previous studies, the voluntary acceptance test has been used either without food (Bernachon et al., 2014a, Cron et al., 2014), with or without food (Bernachon et al., 2014b, Huhtinen et al., 2015), or with food (Gunew et al., 2008). Despite the possible food effects on the bioavailability of the pharmaceutical (Ahmed & Kasraian, 2002), the voluntary acceptance of a pharmaceutical mixed with food is an animal-friendly administration method. However, the product should not be claimed to be palatable if it is accepted only with food, but should rather be defined as ‘acceptable with food’, for example.

We did not have any strict inclusion or exclusion criteria for the cats in this study, in contrast to a study about feeding behaviour assessed by observers where outgoing cat personalities were selected to avoid stress (Van den Bos et al., 2000). Without strict exclusion criteria, a more realistic target population is acquired. The only inclusion criteria in our study were that the cat had a normal appetite and was clinically healthy, according to the owner. However, in hindsight, we should have instructed the owner not to continue the trial if the cat was not motivated to eat the food that was offered. Furthermore, it would have been clearer if the same cats had been used in every trial, particularly in view of the individual taste preferences of the cats.

A few owners submitted comments on the administration of the mini-tablets. For example, a mini-tablet sticking to the cat's nose might reduce the voluntary acceptance even if the mini-tablet was palatable, as cats choose their food based on smell in the first instance (Hullár, Fekete, Andrásofsky, Szöcs, & Berkényi, 2001). Presumably, this could be avoided by offering the mini-tablet by hand. The reason for vomiting after eating the l-carnitine mini-tablet could be related to its unpleasant taste, because no other cat voluntarily accepted it without food. Although not mentioned by the owners, the mini-tablets might be difficult to handle, for example by people with impaired vision or large hands, which might be avoided if a suitable administration device were developed. Moreover, in some cases, the mini-tablet might also be too small to contain an adequate amount of the active drug ingredient, whereupon several tablets would be needed for the proper dosage.

Owner-perceived palatability trials carried out on pet cats in their home environments could be used for testing the palatability of pharmaceuticals for cats. However, due to the heterogenic population, the number of cats should be higher than used in our study (EMA, 2014). Furthermore, the cats should be motivated to eat in each trial, and the owners should be well-instructed. If food is used for concealing the tablet, the foodstuff should be standardized and its palatability verified for each particular cat before the trials. In addition, testing pharmaceuticals in healthy privately owned cats considers an ethical aspect which should be severely considered. Pharmaceuticals tested should not be harmful to the cats.

## 5 Conclusions

Synthetic flavours did not improve the voluntary acceptance of the mini-tablets that were not accepted without food by most of the cats. Hence, the results did not support the earlier literature about amino acids being palatable to cats. The size of the mini-tablet was optimal when concealed inside a food item, and the mouthfeel of the mini-tablet also seemed to be acceptable. The owner-performed palatability tests carried out on pet cats in their home environments could be a feasible method for testing the palatability of pharmaceuticals in cats.

## Declaration of interest

Corporations that have funded the study played no role in the study design nor in the collection, analysis and interpretation of the data, nor in the decision to submit the manuscript for publication. None of the authors of this paper have a financial or personal relationship with other people or organizations that could inappropriately influence or bias the content of the paper.

## Appendix Supplementary materials

Image, application 1
