# Supplementary material for: Acceptability of flavoured pharmaceutically non-active mini-tablets in pet cats tested with a rapid 3-portal acceptance test with and without food
Source: Vet Anim Sci. 2019 Mar 1;7:100054. doi: 10.1016/j.vas.2019.100054 (PMC7386771; doi:10.1016/j.vas.2019.100054)
Supplement: Supplementary file 1 [file mmc1.docx]

**Table S1**

Cats and owners that participated in the trials, and food chosen for the trials.

| Cat number | Owner number | Cat breed | Food chosen for the trials | Trials participated |
| --- | --- | --- | --- | --- |
| 1 | 1 | Abyssinian | Cold cut ham | 2, 4, 5, 6, 7 |
|  |  |  | Minced meat, raw | 3 |
| 2 | 2 | Domestic shorthair | Whiskas anti-Hairball | 1, 2, 3, 4, 6, 7 |
|  |  |  | Whiskas Temptations | 5 |
| 3 | 2 | Maine Coon x Somali cross | Whiskas anti-Hairball/Hill’s oral care dry kibble | 1 |
|  |  |  | Whiskas anti-Hairball/Dried tuna | 2 |
|  |  |  | Whiskas anti-Hairball | 3, 4, 6, 7 |
|  |  |  | Whiskas Temptations | 5 |
| 4 | 2 | Somali | Canned cat food/Hill’s oral care dry kibble | 1 |
|  |  |  | Whiskas anti-Hairball | 2, 3, 4, 6,7 |
|  |  |  | Whiskas Temptations | 5 |
| 5 | 3 | Maine coon | Coshida cat sticks | 2, 3, 4, 5, 6, 7 |
| 6 | 4 | Bengal | Cold cut ham | 1 |
|  |  |  | Almo nature Atlantic ocean tuna | 2, 3, 4, 5 |
|  |  |  | Almo nature Chicken | 6 |
| 7 | 5 | Domestic shorthair | Meatball | 1, 2, 3, 4 |
|  |  |  | Hard cheese | 7 |
| 8 | 5 | Domestic shorthair | Meatball | 1, 2, 3, 4 |
|  |  |  | Hard cheese | 7 |
| 9 | 5 | Domestic shorthair | Meatball | 1, 2, 3, 4 |
|  |  |  | Hard cheese | 7 |
| 10 | 5 | Domestic shorthair | Meatball | 1, 2, 3, 4 |
|  |  |  | Hard cheese | 7 |
| 11 | 6 | Domestic shorthair | Turkey wiener/Canned cat food | 6 |
|  |  |  | Waltham Duck and Rice | 7 |
| 12 | 7 | Abyssinian | Minced meat, raw | 3 |
|  |  |  | Thrive chicken | 5, 6 |
| 13 | 7 | Abyssinian | Dried chicken | 5 |
|  |  |  | Thrive chicken | 6 |
| 14 | 7 | Abyssinian | Minced meat, raw | 3 |
|  |  |  | Thrive chicken | 6 |
| 15 | 8 | Maine Coon x Somali cross | Whiskas anti-Hairball | 5 |
| 16 | 7 | Abyssinian | Minced meat, raw | 3 |
|  |  |  | Hard cheese | 5 |
| 17 | 9 | Burmese | Cold cut ham | 3 |
| 18 | 9 | Burmese | Easy Pill | 3 |
| 19 | 9 | Burmese | Easy Pill | 3 |
| 20 | 10 | Abyssinian | Prima cat canned tuna | 3 |
| 21 | 10 | Bengal | Prima cat canned tuna | 3 |
| 22 | 10 | Somali | Prima cat canned tuna | 3 |
| 23 | 4 | Bengal | Cold cut ham | 1 |
